# Social Activities With Friends and Neighbors in Relation to Cognitive Function in Later Life: The Benefits of Contact and Helping

**DOI:** 10.1177/01640275251379366

**Published:** 2025-09-16

**Authors:** Haejin Jang, Shiyang Zhang, Sae Hwang Han

**Affiliations:** 1 Population Research Center, 12330 University of Texas at Austin, Austin, TX, USA; 2 Department of Human Development & Family Sciences, 12330 University of Texas at Austin, Austin, TX, USA

**Keywords:** informal social activities, friends and neighbors, informal helping, cognitive function, multilevel modeling

## Abstract

Despite a growing body of literature linking social activities to cognitive function, studies specifically examining the potential cognitive benefits of friendships and neighbors remain limited. Drawing on eight waves of nationally representative data from the Health and Retirement Study (1998–2012; *N* = 29,777), we investigated whether two distinct forms of social activities with friends and neighbors—(1) getting together for a social visit and (2) providing informal helping—were associated with cognitive function, which was assessed with a modified version of the Telephone Interview for Cognition Status (m-TICS). Multilevel models provided evidence of within-person associations between both types of social activities (with friends and neighbors) and better cognitive function, with informal helping showing more robust associations. This study highlights the value of an adequate level of contact with friends and neighbors, emphasizing the importance of accessible and meaningful social activities for older adults’ cognitive health.

## Introduction

Declines in cognitive function, including memory, attention, and executive functioning, are common features of aging and are associated with a series of adverse outcomes, including reduced functional abilities, lower quality of life, loss of independence in older adults, and increased mortality (Fratiglioni et al., 2004; Hsu, 2007). While some degree of cognitive decline is a normal part of aging, more pronounced declines can indicate neurodegenerative conditions such as Alzheimer’s disease and other dementias (ADRD), with cognitive impairment often serving as an indicator of disease onset (Marioni et al., 2015). In addition to pharmaceutical and medical interventions, identifying accessible and modifiable lifestyle factors that promote cognitive function at the population level has become a priority in gerontological research.

Among various lifestyle-related strategies for promoting cognitive function, there is growing interest in the role of social participation (Krell-Roesch et al., 2019; Mao et al., 2020). Recognized as a fundamental component of active aging by the World Health Organization (2002), social participation is not only a key determinant of health and well-being in general but is also widely considered as an important target for interventions aimed at promoting cognitive function and protecting against cognitive impairment. While part of the observed association between social participation and health likely reflects selection effects—whereby individuals who engage in activities may already be healthier than those who do not—an accumulating body of theoretical and empirical work suggests that social participation also plays a causal role in supporting better health outcomes, including cognitive function (Umberson & Karas Montez, 2010). Indeed, a growing body of research has demonstrated that older adults with socially integrated lifestyles experience slower cognitive decline (Fratiglioni et al., 2004; Kelly et al., 2017; Li et al., 2021; Zunzunegui et al., 2003). However, much of the existing research has focused on structured forms of social engagement in formal domains, such as the workforce and formal volunteering (for reviews, see Takase et al., 2024; Keefer et al., 2023), or has relied on broad, aggregate index measures of social engagement (for a review, see Piolatto et al., 2022). In contrast, relatively little attention has been given to identifying cognitive benefits unique to specific forms of informal social activities. As such, despite their well-documented importance for mental and physical health, our understanding on how informal social activities shape cognitive functioning in later life remain limited.

This study addresses this gap by examining cognitive outcomes associated with social activities with friends and neighbors. Specifically, we investigate two forms of informal activities involving friends and neighbors— social visits (i.e., getting together with friends and neighbors) and informal helping (i.e., providing help and assistance to friends and neighbors)—to determine their associations with both cognitive function levels and rates of cognitive decline. Using longitudinal data from the *Health and Retirement Study* (HRS), we apply analytic approaches that capture within-person changes in social activities, offering a more dynamic and nuanced perspective on how these everyday interactions shape cognitive trajectories in later life, while also helping to address concerns about selection effects by controlling for stable health and sociodemographic differences across individuals.

### Theoretical Background

There is a strong theoretical foundation supporting the link between social activities and cognitive function. Engaging in various forms of social activities may not only provide direct cognitive stimulation but also expose individuals to an enriched environment that provides opportunities for physical activity, learning, communication skills, and other novel experiences, all of which contribute to maintaining cognitive health in later life (Burr et al., 2021; Keller-Cohen et al., 2006; Li et al., 2021). This view aligns well with cognitive reserve theory, which suggests that social activities may strengthen neural networks and promote cognitive plasticity, enabling individuals to better compensate for age-related cognitive decline and neurological changes by utilizing alternative brain pathways (Scarmeas & Stern, 2003). In addition, social activities often help to cultivate and sustain strong social ties, which in turn provide emotional support, reduce stress, and create further opportunities for meaningful engagement. Through these pathways, frequent participation in social activities may help individuals maintain cognitive function and slow the trajectory of cognitive decline as they age (Berkman et al., 2000; Umberson, 1987).

### Cognitive Outcomes Associated With Social Activities With Friends and Neighbors

The literature indicates that social activities with friends and neighbors can be conceptualized in ways that elucidate their potential links to well-being and cognitive health. Participation in social activities involves the exchange of individual resources through interactions with friends, neighbors, and other social groups (Levasseur et al., 2010). Unlike family and work-related relationships, which may persist regardless of relationship quality, friendships are voluntarily formed and selectively maintained, typically between age-peers with egalitarian dynamics, making them a vital source of companionship and emotional support (Ng et al., 2024; Sharifian et al., 2020). Friends often share similar life experiences, social norms, and lifestyles, making these relationships especially relevant in later life, during which time social activities tend to decline due to factors such as retirement, loss of family members, and reduced mobility (Hartup & Stevens, 1997; Huxhold et al., 2014; Peng et al., 2022). At the same time, neighbors may also play a growing role in later life, particularly due to their physical proximity and availability for more immediate, day-to-day support. While friendships offer emotionally meaningful and voluntarily maintained ties, neighbor relationships, though often weaker, can provide practical forms of connection that become especially relevant as mobility declines (Kalmijn, 2024; Wiles et al., 2012). Given these dynamics, friendships and neighborly interactions may play a critical role in sustaining well-being and health through regular engagement in social activities. However, while earlier studies are suggestive of such health benefits extending to cognitive function, little attention has been paid to how different levels and forms of social activities shape this relationship (Peng et al., 2022).

In this context, a taxonomy proposed by Levasseur and colleagues (2010) provides a useful framework for studying different forms of social participations, which are classified based on (a) levels of involvement with others and (b) the goals of the activity. This framework organizes social activities along a continuum, ranging from preparatory activities (e.g., planning social activities) to more engaged behaviors such as helping others or participating in civic activities. Within this framework, getting together with friends and neighbors (e.g., social visits, casual conversations) represents a moderate level of engagement with others, while informal helping (e.g., assisting with household tasks) is seen as representing a higher level of goal-oriented participation with other people. The present study applies this framework to examine how these two types of informal social activities—getting together and helping—contribute to cognitive function in later life.

Understanding the potential cognitive benefits of informal social activities requires distinguishing between both qualitative and quantitative differences in participation. First, the type and nature of social activity, as described above, may differently influence cognitive outcomes. The literature on social activities suggests that participating in more complex, goal-oriented social activities generally lead to better health outcomes, including longevity, compared to other general and casual forms of social activities (Holt-Lunstad et al., 2010). Specifically, informal helping involves meaningful social activities that may be robustly associated with better cognitive function—akin to the well-documented cognitive benefits of formal volunteering (Nakamura et al., 2024)—as it often requires cognitively stimulating tasks such as problem-solving, planning, and perspective-taking. Beyond cognitive stimulation, helping others may also support cognitive health by providing a protective buffer against chronic stressors commonly experienced in later life, as suggested by broader literature linking helping behaviors to improved health outcomes (Brown & Brown, 2015; Poulin et al., 2013). Although getting together with friends and neighbors may also provide opportunities for social connection and support—both essential for cognitive function—the cognitive benefits of informal helping may be more pronounced, as it often involves greater cognitive engagement as well as stress-buffering effects that are less likely to be elicited by casual social contact.

Second, frequency of participation may also shape how social activities influence cognitive function. Several studies suggest that while social engagement is generally beneficial, its impact varies depending on the dose of engagement (i.e., how often individuals participate). Li and colleagues (2021) observed that frequent social activities with friends enhanced memory, while daily engagement in helping behaviors did not show additional benefits. Krell-Roesch and colleagues (2019) demonstrated that engaging in social activities 1–4 times per week reduced the risk of mild cognitive impairment, but more frequent participation showed diminishing returns. Similarly, Nakamura and colleagues (2024) found that informal helping showed positive associations with cognitive function at both moderate levels (50–99 h per year) and high levels (over 100 h per year) of participation, whereas very low levels of participation yielded no significant benefits. This pattern of findings collectively suggest that it is possible that the link between informal helping and cognition is marked by a threshold effect, as studies focusing on other forms of helping (e.g., volunteering, caregiving) typically indicate that high-intensity engagement may introduce role conflict or stress, thereby diminishing or even reversing its benefits (Kumagai, 2017; Windsor et al., 2008). Taken together, these findings suggest that both the type and frequency of social activities matter, underscoring the need for more precise investigations into how informal social engagement supports cognitive function.

### Research Hypotheses and Overview of the Study

Given the importance of identifying modifiable lifestyle factors that could delay or prevent cognitive impairment in later life, this study focused on investigating cognitive outcomes associated with social activities involving friends and neighbors, an understudied area of research. To this end, we employed longitudinal data collected from a nationally representative sample to examine whether and how two forms of social activities— social visits and informal helping—influence the level of cognitive function and the rate of cognitive decline. We specifically focused on identifying how intra-individual changes in social activities are associated with improvements in cognitive outcomes in an attempt to highlight the modifiable nature of these activities. We hypothesized that both forms of social activities would be associated with better cognitive outcomes, but that the associations would be stronger and more robust for informal helping compared to social visits. We also hypothesized that moderate levels of participation, rather than very low or high levels, would be most strongly associated with cognitive benefits, based on previous evidence suggesting curvilinear effects of social activities.

## Methods

### Data and Study Sample

This study utilized data from the *Health and Retirement Study* (HRS), an ongoing biennial panel survey conducted by the Survey Research Center at the University of Michigan. The HRS was initiated in 1992 with a nationally representative cohort of adults aged 51–61 (born 1931–1941) and their spouses of any age. It was subsequently expanded through sample replenishment to include younger and older birth cohorts, resulting in a comprehensive, nationally representative sample of U.S. adults aged 50 and older (Sonnega et al., 2014). We analyzed eight waves of HRS data collected between 1998 and 2012, a period during which key study variables were measured consistently across waves. Since our study included individuals from new cohorts that were added to the HRS after 1998, “baseline” in this study refers to the first wave at which each individual participated during the observation period. Primary data for this study were drawn from both the RAND-HRS data files and raw data from the original HRS files. The RAND-HRS provided harmonized sociodemographic and health-related variables and addressed inconsistencies and missingness across waves. Several key variables not included in the RAND files—social visits, and informal helping—were constructed using raw variables from the original HRS data.

We selected our analytic sample based on several criteria. The sample was restricted to community living, non-proxy respondents aged 51 and older, who had completed at least one cognitive battery during the study period (*n* = 30,045). We then eliminated about 1% of the sample who had missing data on study variables (*n* = 268). The final analytic sample included 29,777 participants who provided 131,449 person-wave observations, equivalent to about 4.4 observations per participant over the study period.

### Measures

#### Cognitive Function

Cognitive function was evaluated at each survey wave using a modified Telephone Interview for Cognitive Status (m-TICS; Ofstedal et al., 2005), which included four tasks: immediate word recall (range: 0–10), delayed word recall (range: 0–10), serial 7s subtraction (range: 0–5), and backward counting from 20 (range: 0–2). A composite score was created by summing the scores from each task (range: 0–27), with higher scores indicating better cognitive function.

#### Social Activities

At each wave, respondents were asked, “Do you have any good friends living in your neighborhood?” Those respondents who answered “yes” were subsequently asked, “How often do you get together with any of your neighbors just to chat or for a social visit?” We coded the frequency of social visits on a four-point scale (0 = *never*, 1 = *less than weekly*, 2 = *once a week*, 3 = *more than once a week*). Informal helping was assessed by asking, “Have you spent any time in the past 12 months helping friends, neighbors, or relatives who did not live with you and did not pay you for the help?” Respondents who reported engaging in informal helping were the asked about the total annual hours spent helping, which was also recoded to a four-point scale (0 = *never*, 1 = *1–99* *h*, 2 = *100–199* *h*, 3 = *200+* *h*). For both measures, respondents who answered “no” to the initial screening question did not receive the follow-up and were coded as “never” (0). These variables were employed in the analyses as categorical indicators to capture potential threshold or non-linear effects in the associations between informal social activity and cognitive outcomes.

#### Covariates

A set of socio-economic and health characteristics assessed at each wave that could potentially confound the key estimates were included in the analysis, which included marital status (0 = *not married*; 1 = *married*), household wealth (assets minus debts, transformed by the inverse hyperbolic sine-transform function; Friedline et al., 2015), work status (0 = *did not work for pay*; 1 = *worked for pay*), volunteer status (0 = *did not participate in volunteer work*; 1 = *participated in volunteer work*), number of activities of daily living (ADL) limitations (range: 0–5), depressive symptoms measured by the 8-item Center for Epidemiologic Studies Depression (CES-D) scale (range: 0–8), presence of good friends in the neighborhood (0 = *no*; 1 = *yes*), presence of relatives in the neighborhood (0 = *no*; 1 = *yes*), and residential relocation (0 = *did not move*; 1 = *moved between survey waves*). We also included in key demographic characteristics measured at baseline, included age (in years; range: 50–109), years of education (range: 0–17), and race-ethnic status (*non-Hispanic White*, *non-Hispanic Black*, *non-Hispanic other race*, and *Hispanic*), in the analyses.

### Analytic Strategy

We employed multilevel modeling to assess longitudinal associations between informal social activities and cognitive function, with repeated observations (Level 1) nested within individuals (Level 2). To address our research questions, we used within-between random effects (WBRE) models, which separate within-person variation from between-person differences by decomposing time-varying predictors into within-person (WP) and between-person (BP) components (Bell & Jones, 2015). Our primary focus was on the WP effects, which closely approximate estimates from fixed effects models that are unconfounded by any observed or unobserved person-level characteristics. The WP components captured deviations from an individual’s average levels of social activities at a given wave, allowing us to examine how fluctuations in activity were related to cognitive changes over time. In contrast, BP components represented the person’s overall average level of social activities across waves, capturing stable differences between individuals who were more or less socially active.

We followed the best practices recommended in the literature to accurately model trajectories of cognitive change in later life. Given the accelerated longitudinal design of the HRS and the broad age range of cohorts included in the study sample, cognitive change was modeled using both time elapsed from baseline (to capture intra-individual change) and age at baseline (to account for cross-sectional age differences; Morrell et al., 2009). This approach is preferred over using chronological age as the sole time metric, which conflates within-person cognitive aging processes with cohort differences in cognitive function. To account for the non-linear nature of cognitive decline, we included both linear and quadratic terms for time, and further added an interaction term between baseline age and time to account for potential cohort-differences in the rate of decline (Morrell et al., 2009). Finally, we specified random slopes for time to account for substantial inter-individual variability in the rate of cognitive decline.

We present results from three models. First, an unadjusted model (Model 1) was estimated, which included the WP and BP components of the social activity measures, along with the time parameters described above for modeling cognitive trajectories. In Model 2 (i.e., adjusted model), all covariates measured at baseline and over time were added. The research question regarding the association between two forms of social activity and the level of cognitive function was addressed by examining the level-1 WP estimates of each social activity.

To assess whether social activities influence the rate of cognitive decline, Model 3 introduced an interaction term between each form of social activity and time to Model 2. These interaction terms were also decomposed into WP and BP components to avoid bias from stable, unobserved individual characteristics (Schunck, 2013). In all models, the effects of social visits and informal helping were tested simultaneously. Supplementary analyses showed consistent results when each social activity was modeled separately (see Tables S1, S2 in Supplementary Materials). All analyses were conducted using the MIXED function in Stata (Version 18).

## Results

### Study Sample Characteristics

Baseline characteristics of the study sample are presented in Table 1. On average, participants were 62.7 years old, 58.8% were female, and the mean cognitive function score was 15.12 out of 27. A majority of participants engaged in some degree of social visits with friends and neighbors. Specifically, 32.1% reported social visits more than once a week or daily, 21.1% reported once a week, and 29.7% reported less than once a week. In contrast, 17.1% of participants reported no interaction with friends and neighbors in their neighborhood. Compared to social visits, the overall level of engagement in informal helping was relatively low. Nearly half of the sample (45.4%) reported providing no informal helping in the past year. Among those who engaged in informal helping, 37.5% reported providing between 1 and 99 h annually, 10.3% reported 100 and 199 h, and 6.8% providing 200 h or more hours of informal help. The within-person correlation between the two forms of social activity was relatively low (Spearman’s rho = .07), suggesting that social visits and informal helping largely occur independently within the same individuals.

**Table 1.** Descriptive Characteristics of the HRS Sample at Baseline

Variables	Mean/%	(SD)
Cognitive functioning (0–27)	15.12	(4.58)
Social visits (%)		
Never	17.11	
Less than once a week	29.70	
Once a week	21.14	
More than once a week/daily	32.06	
Informal helping (%)		
None	45.40	
1–99 annual hours	37.47	
100–199 annual hours	10.28	
200+ annual hours	6.84	
Female (%)	58.80	
Married or partnered (%)	61.31	
Education (years, 0–17)	12.43	(3.22)
Working for pay (%)	36.81	
Household wealth (IHS-transformed)	4.98	(2.62)
ADL limitations (0–5)	0.29	(0.80)
Depressive symptoms (CES-D, 0–8)	1.51	(1.97)
Volunteering status (yes, %)	34.50	
Race/ethnicity (%)		
Non-Hispanic White	73.35	
Non-Hispanic Black	15.07	
Hispanic	9.32	
Non-Hispanic other	2.27	

*Note.* Sample *N* = 29,777. Time-varying variables reflect person-wave averages.

ADL = activities of daily living; CES-D = center for epidemiologic studies–depression scale; IHS = inverse hyperbolic sine; m-TICS = modified telephone interview for cognitive status.

### Multilevel Model Results

Results from the multilevel models based on WBRE approach are presented in Table 2. The WP effects of linear and quadratic time were statistically significant, indicating a curvilinear pattern of cognitive decline over time. In addition, the random variance estimates for both the intercept and time were significant, reflecting substantial inter-individual variability in the baseline level of cognitive function and the rate of cognitive decline.

**Table 2.** Multilevel Model Results Linking Social Activities and Cognitive Function Levels

Variables	Model 1: Unadjusted		Model 2: Adjusted	
	Estimate	(SE)	Estimate	(SE)
Fixed effects				
*Within-person effects (level 1)*				
Linear time	−0.322***	(0.012)	−0.315***	(0.012)
Quadratic time	−0.018***	(0.002)	−0.017***	(0.002)
Social visits				
Less than once a week	0.060*	(0.030)	0.049	(0.035)
Once a week	0.066*	(0.033)	0.051	(0.037)
More than once a week/daily	0.102**	(0.032)	0.086*	(0.037)
Informal helping				
1–99 annual hours	0.133***	(0.023)	0.107***	(0.023)
100–199 annual hours	0.218***	(0.033)	0.193***	(0.033)
200+ annual hours	0.174***	(0.040)	0.153***	(0.040)
Married			−0.091*	(0.044)
Household wealth (IHS-transformed)			0.035***	(0.006)
Working for pay			0.125***	(0.030)
ADL limitations			−0.218***	(0.016)
Volunteering			0.124***	(0.026)
Depressive symptoms			−0.050***	(0.006)
Having good friends in neighborhood			−0.008	(0.028)
Having relatives in neighborhood			0.028	(0.026)
Residential relocation			−0.056	(0.032)
*Between-person effects (level 2)*				
Linear time	0.593***	(0.073)	0.141*	(0.061)
Quadratic time	−0.040**	(0.013)	−0.002	(0.011)
Social visits				
Fewer than once a week	0.504***	(0.087)	0.240*	(0.093)
Once a week	0.625***	(0.095)	0.287**	(0.100)
More than once a week/daily	−0.233**	(0.082)	0.118	(0.094)
Informal helping				
1–99 annual hours	2.725***	(0.067)	0.834***	(0.061)
100–199 annual hours	2.994***	(0.118)	0.979***	(0.102)
200+ annual hours	3.541***	(0.133)	1.447***	(0.114)
Married			−0.272	(0.041)
Household wealth (IHS-transformed)			0.131***	(0.008)
Working for pay			0.767***	(0.052)
ADL limitations			−0.166***	(0.027)
Volunteering			0.638***	(0.050)
Depressive symptoms			−0.205***	(0.012)
Have good friends in neighborhood			−0.405***	(0.068)
Have relatives in neighborhood			−0.207***	(0.048)
Residential relocation			0.033	(0.107)
*Background characteristics (level 2)*				
Linear age	−0.145***	(0.004)	−0.111***	(0.004)
× linear time	−0.023***	(0.001)	−0.022***	(0.001)
Quadratic age	−0.001**	(0.000)	−0.002***	(0.000)
Female			0.931***	(0.036)
Race-ethnic status, %				
Black (non-Hispanic)			−2.177***	(0.049)
Others (non-Hispanic)			−1.240***	(0.106)
Hispanic (any race)			−0.679***	(0.061)
Education in years			0.382***	(0.006)
Intercept	12.270***	(0.082)	13.476***	(0.097)
Random effects				
Time variance	0.102***	(0.004)	0.097***	(0.004)
Intercept variance	10.482***	(0.120)	6.608***	(0.088)
Cov (time, intercept)	−0.258***	(0.019)	−0.255***	(0.016)
Residual variance	6.831***	(0.033)	6.833***	(0.033)
Model fit				
−2 log-likelihood	686,780.292		675,120.364	
AIC	686,830.291		675,216.364	

*Note.* Sample *N* = 29,777. Person-wave observation *N* = 131,449.

IHS = inverse hyperbolic sine; ADL = activities of daily living.

*p < .05. **p < .01. ***p < .001.

**Figure 1.**
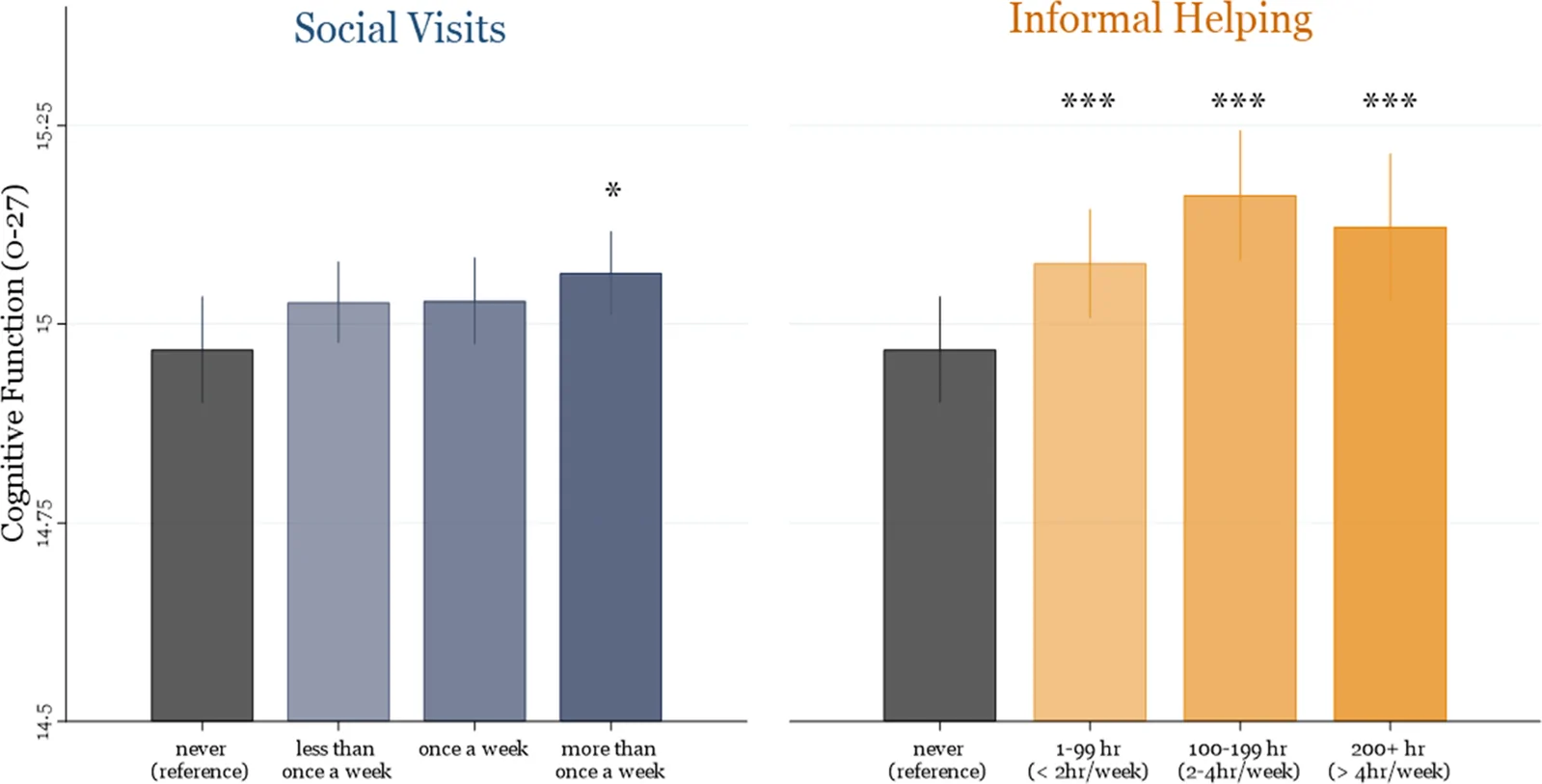
Within-Person Associations Between Social Activities and Cognitive Functioning. Plots are Based on Estimates Presented in Model 2, Table 2. Asterisks Indicate Statistically Significant Differences From the Reference Category (“Never”). *p < .05, ***p < .001

WP estimates of social visits were all positive and statistically significant, indicating that individuals reported higher levels of cognitive function during periods when they engaged in social visits with friends and neighbors compared to when they did not. In the adjusted model, however, this positive association was only observed when social visits took place more than once a week or daily (*b* = 0.086, *p* = .02), with no significant WP effects for less frequent visits. In contrast, informal helping demonstrated robust and consistent WP associations with cognitive function, in both unadjusted and adjusted models. That is, even relatively low involvement (1–99 annual hours) was associated with a higher level of cognitive function, even after adjusting for time-varying covariates in Model 2 (*b* = 0.108, *p* < .001). Similar positive associations were observed for moderate (100–199 hours: *b* = 0.193, *p* < .001) and high levels of engagement (200+ hours: *b* = 0.153, *p* < .001). When we re-estimated the models using the lowest-dose level of each activity as the reference category (i.e., less than once a week for social visits; 1–99 h for informal helping), we found no statistically significant differences across social visit frequencies. However, a moderate level of informal helping (100–199 h) conferred additional cognitive benefit compared to the 1–99 h of helping (*b* = 0.085, *p* = .005; full results available upon request). These WP findings are summarized in Figure 1.

Although our research questions focused primarily on WP associations, the BP associations between social activities and cognitive function offer useful context for understanding the findings. On the one hand, individuals who, on average, engaged in more informal helping over the study period exhibited higher levels of cognitive function compared to those who did not, in a manner that reflected a dose-response relationship. That is, compared to individuals who never engaged in informal helping, those who consistently reported 200+ h, 100–199 h, and 1–99 h of informal helping had higher cognitive function scores by 1.470 (*p* < .001), 0.844 (*p* < 0.01), and 0.613 (*p* < .001), respectively, (see Model 2, Table 2). On the other hand, such BP differences were observed only for individuals who reported social visits fewer than once a week (*b* = 0.187, *p* = .047) or once a week (*b* = 0.204, *p* = .043) compared to who did not report any social visits, but not for those who reported social visits more than once a week or daily (*b* = 0.009, *p* = .922).

**Figure 2.**
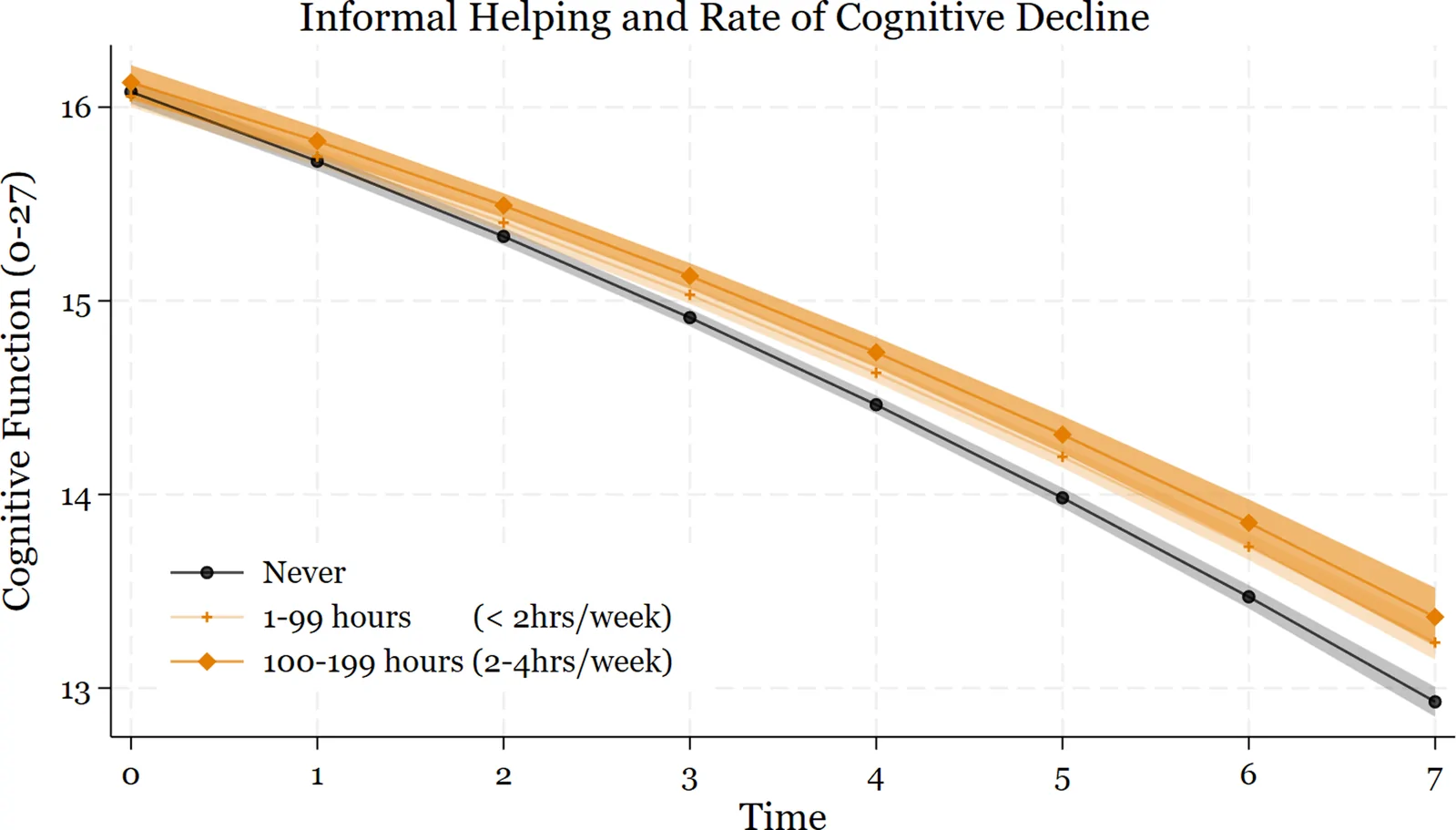
Trajectories of Cognitive Function Over Time by Levels of Informal Helping, Based on Within-Person Estimates From Model 3 (Table 3). The Plots Illustrate Hypothetical Cognitive Decline Trajectories for Individuals Under a Scenario of no Engagement in Informal Helping, Compared to Trajectories for the Same Individuals Under Scenarios of Consistent Engagement in 1–99 or 100–199 h of Helping Annually (200+ h Not Shown)

The model results on the linkages between social activities and the rate of cognitive decline are presented in Model 3, Table 3 (see also Figure 2). While social visits were unrelated to the rate of cognitive decline, we observed a slower rate of cognitive decline when respondents engaged in 1–99 h (*b* = 0.047, *p* < .001) and 100–99 h (*b* = 0.056, *p* < .001) of informal helping compared to when they did not engage in helping. Such effects were not observed for 200+ h of informal helping (*b* = 0.022, *p* = .232).

**Table 3.** Multilevel Model Results Linking Social Activities and the Rate of Cognitive Decline

Variables	Model 2: Adjusted (copied from Table 2)		Model 3: Interaction	
	Estimate	(SE)	Estimate	(SE)
Fixed effects				
*Within-person effects (level 1)*				
Linear time	−0.315***	(0.012)	−0.350***	(0.016)
Social visits				
Less than once a week	0.049	(0.035)	0.021	(0.049)
Once a week	0.051	(0.037)	0.007	(0.053)
More than once a week/daily	0.086*	(0.037)	0.120*	(0.051)
Informal helping				
1–99 annual hours	0.107***	(0.023)	−0.023	(0.035)
100–199 annual hours	0.193***	(0.033)	0.048	(0.050)
200+ annual hours	0.153***	(0.040)	0.088	(0.057)
Linear time × social visits				
Less than once a week			0.010	(0.013)
Once a week			0.016	(0.014)
More than once a week/daily			−0.013	(0.013)
Linear time × informal helping				
1–99 annual hours			0.047***	(0.009)
100–199 annual hours			0.056***	(0.015)
200+ annual hours			0.022	(0.018)
Intercept	13.476***	(0.097)	13.553***	(0.108)
Random effects				
Time variance	0.097***	(0.004)	0.097***	(0.004)
Intercept variance	6.608***	(0.088)	6.607***	(0.088)
Cov (time, intercept)	−0.255***	(0.016)	−0.255***	(0.016)
Residual variance	6.833***	(0.033)	6.832***	(0.033)
Model fit				
−2 log-likelihood	675,120.364		675,069.818	
AIC	675,216.364		675,189.819	

*Note.* Sample *N* = 29,777. Person-wave observation *N* = 131,449.

IHS = inverse hyperbolic sine; ADL = activities of daily living.

Model estimates adjusted for the full set of covariates listed in Table 2.

*p < .05. ***p < .001.

## Discussion

In the context of population aging and rising concerns about the prevalence of cognitive impairment and related dementias, identifying modifiable lifestyle factors that promote cognitive health has become increasingly critical. While previous research has highlighted the benefits of social engagement, relatively few studies have distinguished between specific forms and frequencies of social activity in relation to cognitive outcomes. Addressing this gap, this study examined cognitive outcomes associated with two forms of social activities—social visits and informal helping. Drawing on eight waves of longitudinal data from the *Health and Retirement Study* (HRS), we investigated whether engagement in these informal social activities is associated with higher cognitive function and a slower rate of cognitive decline. Findings from a within-person analytic approach indicate that both types of social activities with friends and neighbors are linked to better cognitive function, with informal helping showing particularly robust associations.

Findings from this study provide novel insights into how social visits and informal helping may contribute to cognitive function in later life. On the one hand, social visits with friends and neighbors were associated with higher levels of cognitive function, but this association was observed only at higher frequencies. That is, cognitive function was positively affected by social visits only when they occurred at least weekly or daily, but not below this threshold, suggesting that less frequent, occasional social visits are insufficient to provide sustained cognitive engagement or stimulation needed to support cognitive function. Additionally, social visits were unrelated to the rate of cognitive decline, suggesting that while frequent social visits may provide immediate cognitive benefits, they do not necessarily protect against long-term cognitive decline. On the other hand, informal helping was consistently associated with both cognitive function levels and rates of cognitive decline. In contrast to social visits, any engagement in informal helping was linked to better cognitive function, but notably, low to moderate levels of helping showed the strongest protective effect against cognitive decline. Our findings indicate robust within-person associations between both forms of informal social activities and cognitive functioning. This aligns with previous research demonstrating positive associations between informal social activities (e.g., informal helping, visiting friends) and cognitive function (Huxhold et al., 2014; Li et al., 2021; Nakamura et al., 2024). However, the insufficient presence of a within-person analytical approach in previous studies makes it difficult to determine whether changes in an individual’s own level of social engagement over time are associated with cognitive trajectories.

### Types of Social Activities and Cognitive Function

The stronger cognitive benefits of informal helping compared to social visits may be partly explained by the complexity and nature of engagement (Levasseur et al., 2010). Engaging in meaningful and purposeful social roles is known to have a more significant impact on long-term health outcomes (Holt-Lunstad et al., 2010). Social visits primarily foster social bonding and emotional support, providing a moderate level of interaction, whereas informal helping entails a higher level of goal-directed engagement (Levasseur et al., 2010). While social visits may involve passive participation and casual conversations with limited cognitive stimulation, informal helping requires intentional and active involvement. For example, assisting with household tasks extends beyond simple social activities. It requires identifying others’ needs, planning appropriate actions, and carrying them out—engaging multiple cognitive processes. Unlike passive social activities, these goal-oriented activities demand continuous cognitive engagement, actively stimulate multiple domains of cognitive function, and may contribute to long-term cognitive benefits.

Moreover, unlike structured social activities (e.g., formal volunteering), informal helping is naturally embedded in daily life and often demands flexible problem-solving, self-regulation, and adaptive thinking (Einolf et al., 2016; Martinez et al., 2011). This dynamic and unstructured nature requires individuals to continuously assess needs, navigate varying social contexts, and make real-time decisions, thereby potentially promoting better cognitive function. While our study is consistent with earlier findings linking informal helping and better cognitive outcomes (Nakamura et al., 2024), the underlying mechanisms remain unclear. It is also possible that the neurobiological mechanisms underlying the prosocial aspects of informal helping—such as hormonal regulation, activation of brain regions associated with empathy and social cognition, and stress-buffering effects—may contribute to both short- and long-term cognitive benefits (Brown et al., 2012). Further research is needed to elucidate these pathways and their implications for cognitive aging.

### Frequency of Social Activities and Cognitive Function

Another noteworthy finding of this study is that high levels of informal helping (i.e., more than 200 h per year, equivalent to 4 or more weekly hours) did not necessarily lead to more cognitive benefits compared to smaller doses of helping on both the within- and between-person levels. One explanation is that a high level of commitment to helping in the community may lead to role conflict, where individuals experience heightened stress due to competing responsibilities (Thoits, 2012). Alternatively, burnout, physical fatigue, or increased social burden may contribute to this pattern, as prolonged engagement in helping activities could shift from a rewarding experience to a chronic stressor. These findings suggest that there may be an optimal level of social engagement that maximizes cognitive benefits while minimizing potential negative consequences.

It is important to note that the findings reported here represent average effects across the population. While such estimates are important and informative, they may mask important differences across sociodemographic subgroups. Prior research suggests that both social relationships and cognitive function in later life are gendered, and that risks of social isolation and cognitive impairment are unequally distributed across sociodemographic lines (Levine et al., 2021; Umberson & Donnelly, 2023). To address the potential effect heterogeneity across subgroups, we conducted elaboration analyses by gender, race-ethnicity, and marital status. Gender differences were limited, suggesting that men and women, on average, experience similar cognitive benefits from the two forms of social activities (see Table S3 in Supplementary Materials). However, we found that marital status moderated the association between social visits and cognitive function among men, with married men showing stronger cognitive benefits compared to their unmarried counterparts (see Tables S4, S5 in Supplementary Materials). One possible explanation is that men may experience more meaningful and cognitively enriching social interactions when embedded in spousal or spousal-linked networks. Further research is needed to clarify the mechanisms through which married men benefit from social visits and, importantly, to identify the types and contexts of social engagement that may support cognitive health among unmarried men in later life.

### Limitations

Despite its contributions, this study has several limitations. First, utilizing secondary data from the HRS inherently constrained the methodological approach taken in this study, particularly in terms of measurement specificity and contextual detail. For example, the HRS lacked detailed information on social activities, as it did not capture the context or nature of social visits, nor did it distinguish between interactions involving friends versus neighbors. Similarly, the informal helping measure did not differentiate between types of recipients (e.g., friends, neighbors, and relatives) or motivations for helping. As such, it is possible that some of the cognitive outcomes associated with helping were confounded by unmeasured interactions with kin that may independently influence cognitive health. To partly address this issue, we reanalyzed the models with additional controls for the number of siblings and children (see Table S6 in Supplementary Materials), and the results were consistent with the main findings. Still, these limitations hindered our ability to fully isolate the unique cognitive effects corresponding to specific types and the nature of social visits and helping behaviors with friends and neighbors. Second, although the WBRE approach helped to alleviate omitted variable bias stemming from all time-invariant characteristics, the WP estimates still are subject to bias from unmeasured sources of time-varying confounders, which prevented us from discussing the findings in causal terms. For example, certain changes in neighborhood characteristics—such as shifts in socioeconomic conditions or exposure to natural disasters (Besser et al., 2017)—could simultaneously affect both individuals’ opportunities for social engagement and their cognitive health, thereby partly confounding the observed associations. Third, the findings have limited generalizability to contexts outside of the US, where cultural norms around friendship, neighbors, helping, and aging may differ. Cross-national research is needed to better understand how social activities relate to cognitive health across diverse cultural and policy contexts.

## Conclusion

This study extends previous research by empirically testing whether social activities in the informal domain can function as a modifiable health behavior that mitigates cognitive decline in later life. While previous studies have often focused on structured activities, such as formal volunteering and labor force participation, and relied on aggregated measures of social engagement, this study focused on two unique forms of informal social activities, finding that cognitive benefits depend not only on the frequency of social activities but also on the type of participation with friends and neighbors. While frequent social visits are linked to higher cognitive function, their long-term benefits appear limited without meaningful, cognitively engaging content. In contrast, informal helping shows consistent associations with both higher cognitive function and slower cognitive decline, particularly at low to moderate levels of engagement. Identifying other forms of social activities (e.g., caregiving for grandchildren, attending local community groups, educational/training courses) that may confer similar cognitive benefits presents a fruitful area of future research.

These findings highlight the importance of fostering not just more, but more purposeful social activity among older adults. As digital communications and passive leisure activities (e.g., screen time) become more prevalent, time spent engaging in cognitively enriching activities is becoming increasingly limited. Therefore, public health strategies should consider prioritizing informal social activities that are mentally stimulating, personally meaningful, and sustainable, rather than simply focusing on increasing social contact. Encouraging and providing opportunities for informal helping may offer a promising, low-cost avenue for promoting cognitive health and overall well-being in aging populations (Han et al., 2025).

## Supplemental Material

Supplemental Material - Social Activities with Friends and Neighbors in Relation to Cognitive Function in Later Life: The Benefits of Contact and Helping Supplemental Material for Social Activities with Friends and Neighbors in Relation to Cognitive Function in Later Life: The Benefits of Contact and Helping by Haejin Jang, Shiyang Zhang, and Sae Hwang Han in Research on Aging

## Data Availability

The data used in this study are from the Health and Retirement Study (HRS), sponsored by the National Institute on Aging (grant number NIA U01AG009740) and conducted by the University of Michigan. The publicly available data are accessible through the Institute for Social Research at the University of Michigan: https://hrsdata.isr.umich.edu/data-products/public-survey-data.
