# Supplementary material for: Social Activities With Friends and Neighbors in Relation to Cognitive Function in Later Life: The Benefits of Contact and Helping
Source: Res Aging. 2025 Sep 16;48(9-10):458–70. doi: 10.1177/01640275251379366 (PMC13499910; doi:10.1177/01640275251379366)
Supplement: Supplementary file 1 [file sj-pdf-1-roa-10.1177_01640275251379366.pdf]

Table S1

Multilevel Model Results Linking Social Visits and Cognitive Function Levels

Variables	Unadjusted		Adjusted	
	Estimate	(*SE*)	*Estimate*	(*SE*)
**Fixed effects**				
Within-person effects (Level 1)				
Linear time	-0.328***	(0.012)	-0.320***	(0.012)
Quadratic time	-0.018***	(0.002)	-0.017***	(0.002)
Social visits				
Less than once a week	0.065*	(0.030)	0.050	(0.035)
Once a week	0.075*	(0.033)	0.056	(0.037)
More than once a week/daily	0.116***	(0.032)	0.095*	(0.037)
Married			-0.096*	(0.044)
Household wealth (IHS-transformed)			0.035***	(0.006)
Working for pay			0.123***	(0.030)
ADL limitations			-0.221***	(0.016)
Volunteering			0.136***	(0.026)
Depressive symptoms			-0.050***	(0.006)
Having good friends in neighborhood			-0.005	(0.028)
Having relatives in neighborhood			0.032	(0.026)
Residential relocation			-0.058	(0.032)
Background characteristics (Level 2)				
Linear age	-0.177 ***	(0.004)	-0.118***	(0.004)
× Linear time	-0.024***	(0.001)	-0.022***	(0.001)
Quadratic age	-0.001***	(0.000)	-0.002***	(0.000)
Female			0.875***	(0.035)
Race-ethnic status, %				
Black (non-Hispanic)			-2.223***	(0.049)
Others (non-Hispanic)			-1.288***	(0.107)
Hispanic (any race)			-0.818***	(0.061)
Education in years			0.394***	(0.006)
Intercept	13.121***	(0.083)	13.815***	(0.095)
**Random effects**				
Time variance	0.103***	(0.004)	0.098***	(0.004)
Intercept variance	11.435***	(0.120)	6.692***	(0.089)
Cov (Time, Intercept)	-0.240***	(0.019)	-0.255***	(0.016)
Residual variance	6.828***	(0.033)	6.834***	(0.033)
**Model fit**				
−2 log-likelihood	689,187.754		675,482.626	
AIC	689,225.755		675,566.627	

Notes. Sample N = 29,777. Person-wave observation N = 131,449. IHS = inverse hyperbolic sine. ADL = activities of daily living. *$p < .05$. ***$p < .001$.

Table S2

Multilevel Model Results Linking Informal Helping and Cognitive Function Levels

Variables	Unadjusted		Adjusted	
	Estimate	(*SE*)	*Estimate*	(*SE*)
**Fixed effects**				
Within-person effects (Level 1)				
Linear time	-0.323***	(0.012)	-0.316***	(0.012)
Quadratic time	-0.018***	(0.002)	-0.017***	(0.002)
Informal helping				
1-99 annual hours	0.138***	(0.023)	0.110***	(0.023)
100-199 annual hours	0.223***	(0.033)	0.195***	(0.033)
200+ annual hours	0.178***	(0.039)	0.155***	(0.040)
Married			-0.093*	(0.044)
Household wealth (IHS-transformed)			0.035***	(0.006)
Working for pay			0.123***	(0.030)
ADL limitations			-0.219***	(0.016)
Volunteering			0.125***	(0.026)
Depressive symptoms			-0.050***	(0.006)
Having good friends in neighborhood			0.020	(0.024)
Having relatives in neighborhood			0.029	(0.026)
Residential relocation			-0.056	(0.032)
Background characteristics (Level 2)				
Linear age	-0.147***	(0.004)	-0.111***	(0.004)
× Linear time	-0.023***	(0.001)	-0.022***	(0.001)
Quadratic age	-0.001*	(0.000)	-0.002***	(0.000)
Female			0.930***	(0.036)
Race-ethnic status, %				
Black (non-Hispanic)			-2.175***	(0.049)
Others (non-Hispanic)			-1.239***	(0.106)
Hispanic (any race)			-0.681***	(0.061)
Education in years			0.383***	(0.006)
Intercept	12.463***	(0.065)	13.560***	(0.089)
**Random effects**				
Time variance	0.102***	(0.004)	0.097***	(0.004)
Intercept variance	10.530***	(0.120)	6.610***	(0.088)
Cov (Time, Intercept)	-0.256***	(0.019)	-0.255***	(0.016)
Residual variance	6.831***	(0.033)	6.833***	(0.033)
**Model fit**				
−2 log-likelihood	686,933.684		675,138.484	
AIC	686,971.685		675,222.484	

Notes. Sample N = 29,777. Person-wave observation N = 131,449. IHS = inverse hyperbolic sine. ADL = activities of daily living. *$p < .05$. ***$p < .001$.

Table S3

Social Activity Effects on Cognitive Function by Gender

Variables	Model A: Main effects (Copied from Table 2)		Model B: Gender interaction	
	Estimate	(*SE*)	*Estimate*	(*SE*)
**Fixed effects**				
Within-person effects (Level 1)				
Social visits				
Less than once a week	0.049	(0.035)	0.047	(0.051)
Once a week	0.051	(0.037)	-0.007	(0.054)
More than once a week/daily	0.086*	(0.037)	0.022	(0.054)
Informal helping				
1-99 annual hours	0.107***	(0.023)	0.080*	(0.035)
100-199 annual hours	0.193***	(0.033)	0.133**	(0.051)
200+ annual hours	0.153***	(0.040)	0.088	(0.067)
Gender × Social visits				
Less than once a week			0.043	(0.045)
Once a week			0.097	(0.066)
More than once a week/daily			0.100	(0.082)
Gender × Informal helping				
1-99 annual hours			0.000	(0.061)
100-199 annual hours			0.099	(0.066)
200+ annual hours			0.109	(0.066)
**Model fit**				
−2 log-likelihood	675,120.364		675,110.686	
AIC	675,216.364		675,218.687	

Notes. Sample N = 29,777. Person-wave observation N = 131,449. Model estimates adjusted for the full set of covariates listed in Table 2.
*$p < .05$. **$p < .01$. ***$p < .001$.

Table S4

Social Activity Effects on Cognitive Function by Marital Status: Women

Variables	Model A: Main effects		Model B: Marital status interaction	
	Estimate	(*SE*)	*Estimate*	(*SE*)
**Fixed effects**				
Within-person effects (Level 1)				
Social visits				
Less than once a week	0.045	(0.046)	0.087	(0.061)
Once a week	0.086	(0.049)	0.167*	(0.066)
More than once a week/daily	0.125*	(0.050)	0.138*	(0.063)
Informal helping				
1-99 annual hours	0.100***	(0.030)	0.144***	(0.044)
100-199 annual hours	0.199***	(0.043)	0.245***	(0.064)
200+ annual hours	0.152**	(0.050)	0.171*	(0.076)
Marital status × Social visits				
Less than once a week			-0.086	(0.077)
Once a week			-0.155	(0.085)
More than once a week/daily			-0.032	(0.082)
Marital status × Informal helping				
1-99 annual hours			-0.082	(0.058)
100-199 annual hours			-0.085	(0.085)
200+ annual hours			-0.037	(0.099)
**Model fit**				
−2 log-likelihood	400,742.642		400,728.046	
AIC	400,836.641		400,846.045	

Notes. Sample *N* = 29,777. Person-wave observation *N* = 77,293. Model estimates adjusted for the full set of covariates listed in Table 2.

*$p < .05$. **$p < .01$. ***$p < .001$.

Table S5

Social Activity Effects on Cognitive Function by Marital Status: Men

Variables	Model A: Main effects		Model B: Marital status interaction	
	Estimate	(*SE*)	*Estimate*	(*SE*)
**Fixed effects**				
Within-person effects (Level 1)				
Social visits				
Less than once a week	0.046	(0.052)	-0.137	(0.090)
Once a week	-0.005	(0.055)	-0.162	(0.097)
More than once a week/daily	0.022	(0.055)	-0.156	(0.092)
Informal helping				
1-99 annual hours	0.122***	(0.034)	0.157*	(0.065)
100-199 annual hours	0.189***	(0.050)	0.063	(0.098)
200+ annual hours	0.154*	(0.064)	0.208	(0.125)
Marital status × Social visits				
Less than once a week			0.254*	(0.101)
Once a week			0.224*	(0.109)
More than once a week/daily			0.253*	(0.104)
Marital status × Informal helping				
1-99 annual hours			-0.044	(0.074)
100-199 annual hours			0.161	(0.112)
200+ annual hours			-0.073	(0.143)
**Model fit**				
−2 log-likelihood	273,853.692		273,834.166	
AIC	273,947.692		273,952.166	

Notes. Sample N = 29,777. Person-wave observation N = 54,156. Model estimates adjusted for the full set of covariates listed in Table 2.
*p < .05. ***p < .001.

Table S6

Sensitivity Analysis: Models with Additional Kinship Network Controls

Variables	Model A (Copied from Table 2)		Model B Extended covariates	
	Estimate	(*SE*)	*Estimate*	(*SE*)
**Fixed effects**				
Within-person effects (Level 1)				
Linear time	-0.315***	(0.012)	-0.319***	(0.012)
Quadratic time	-0.017***	(0.002)	-0.016***	(0.002)
Social visits				
Less than once a week	0.049	(0.035)	0.051	(0.035)
Once a week	0.051	(0.037)	0.056	(0.037)
More than once a week/daily	0.086*	(0.037)	0.088*	(0.037)
Informal helping				
1-99 annual hours	0.107***	(0.023)	0.104***	(0.023)
100-199 annual hours	0.193***	(0.033)	0.192***	(0.033)
200+ annual hours	0.153***	(0.040)	0.143***	(0.040)
Number of children			0.040*	(0.018)
Number of siblings			-0.007	(0.014)
Married	-0.091*	(0.044)	-0.091*	(0.045)
Household wealth (IHS-transformed)	0.035***	(0.006)	0.037***	(0.006)
Working for pay	0.125***	(0.030)	0.125***	(0.031)
ADL limitations	-0.218***	(0.016)	-0.221***	(0.016)
Volunteering	0.124***	(0.026)	0.129***	(0.026)
Depressive symptoms	-0.050***	(0.006)	-0.048***	(0.006)

Having good friends in neighborhood	-0.008	(0.028)	-0.011	(0.028)
Having relatives in neighborhood	0.028	(0.026)	0.026	(0.026)
Residential relocation	-0.056	(0.032)	-0.055	(0.033)
**Model fit**				
−2 log-likelihood	675,120.364		663,825.872	
AIC	675,216.364		663,929.871	
N	131,449		129,267	

Notes. Sample *N* = 29,777. IHS = inverse hyperbolic sine. ADL = activities of daily living. Model estimates adjusted for the full set of covariates listed in Table 2.
**p* < .05. ****p* < .001.
